# KairoSight: Open-Source Software for the Analysis of Cardiac Optical Data Collected From Multiple Species

**DOI:** 10.3389/fphys.2021.752940

**Published:** 2021-10-29

**Authors:** Blake L. Cooper, Chris Gloschat, Luther M. Swift, Tomas Prudencio, Damon McCullough, Rafael Jaimes, Nikki G. Posnack

**Affiliations:** ^1^Sheikh Zayed Institute for Pediatric Surgical Innovation, Children’s National Hospital, Washington, DC, United States; ^2^Children’s National Heart Institute, Children’s National Hospital, Washington, DC, United States; ^3^Department of Pharmacology and Physiology, George Washington University, Washington, DC, United States; ^4^Department of Pediatrics, George Washington University, Washington, DC, United States

**Keywords:** optical mapping of calcium and action potentials, cardiac electrophysiology, Langendorff model, excitation contraction coupling, human induced pluripotent stem cell derived cardiomyocytes, optocardiography, python, species differences

## Abstract

Cardiac optical mapping, also known as optocardiography, employs parameter-sensitive fluorescence dye(s) to image cardiac tissue and resolve the electrical and calcium oscillations that underly cardiac function. This technique is increasingly being used in conjunction with, or even as a replacement for, traditional electrocardiography. Over the last several decades, optical mapping has matured into a “gold standard” for cardiac research applications, yet the analysis of optical signals can be challenging. Despite the refinement of software tools and algorithms, significant programming expertise is often required to analyze large optical data sets, and data analysis can be laborious and time-consuming. To address this challenge, we developed an accessible, open-source software script that is untethered from any subscription-based programming language. The described software, written in python, is aptly named “*KairoSight*” in reference to the Greek word for “opportune time” (Kairos) and the ability to “see” voltage and calcium signals acquired from cardiac tissue. To demonstrate analysis features and highlight species differences, we employed experimental datasets collected from mammalian hearts (Langendorff-perfused rat, guinea pig, and swine) dyed with RH237 (transmembrane voltage) and Rhod-2, AM (intracellular calcium), as well as human induced pluripotent stem cell-derived cardiomyocytes (hiPSC-CM) dyed with FluoVolt (membrane potential), and Fluo-4, AM (calcium indicator). We also demonstrate cardiac responsiveness to ryanodine (ryanodine receptor modulator) and isoproterenol (beta-adrenergic agonist) and highlight regional differences after an ablation injury. *KairoSight* can be employed by both basic and clinical scientists to analyze complex cardiac optical mapping datasets without requiring dedicated computer science expertise or proprietary software.

## Introduction

Cardiovascular research has been advanced by the development of parameter-sensitive dyes, which can be used to monitor transmembrane voltage (V_m_) and intracellular calcium (Ca^2+^) within cardiac tissue (Jaimes et al., 2019a). Fluorescent reporters allow researchers to monitor action potentials and calcium transients in excitable cells and identify underlying alterations in ionic currents. Forty years ago, Salama and Morad acquired the first optical action potentials from heart muscle using Merocyanine-540 (Salama and Morad, 1976), and a decade later, Lee et al. (1987) directly measured intracellular calcium transients from heart preparations using the cytosolic calcium indicator Indo-1, AM. A variety of parameter-sensitive probes have since been incorporated into cardiac research, including ratiometric (e.g., Fura-2/4/8, Indo-1) and non-ratiometric calcium probes (e.g., Rhod-2/4, BAPTA-1, Fluo-2/3/4/8, Cal-520/590/630, Calbryte-520/590/630), and voltage sensitive dyes (e.g., RH237, Di-4-ANEPPS, Di-8-ANEPPS, Di-4-ANBDQPQ, Di-ANBDQBS, PGH-1, FluoVolt). Complementary probes can be used in dual-imaging approaches to simultaneously monitor action potentials and intracellular calcium handling (Choi and Salama, 2000; Salama and Hwang, 2009; Jaimes et al., 2019a). In optical mapping approaches, cardiac tissue preparations (or cells) are stained to monitor dynamic changes in fluorescent emission using scientific cameras with high spatiotemporal resolution. This offers the advantage of increased spatial resolution, compared with recordings from electrode arrays (Fluhler et al., 1985). Recent advances in cardiac imaging applications include: the development of probes with enhanced signal-to-noise characteristics or red-shifted dyes (Matiukas et al., 2007; Lee et al., 2019; Martišienė et al., 2020) to increase imaging depth, incorporation of mechanical uncouplers or motion tracking to reduce/remove motion artifact (Fedorov et al., 2007; Bourgeois et al., 2011; Jaimes et al., 2016b; Zhang et al., 2016; Kappadan et al., 2020), development of panoramic imaging techniques (Bray et al., 2000; Kay et al., 2004; Lee et al., 2017; Gloschat et al., 2018), and improvements in photodetector and light source technology (Grinvald et al., 1981; Fillette and Nassif, 1987; Salama et al., 1987).

To date, cardiac optical mapping, also known as optocardiography (Boukens and Efimov, 2014; George and Efimov, 2019; Swift et al., 2019), has been used to investigate changes in electrical activity, spiral wave formation (Jalife, 2003), excitation-contraction coupling (Christoph et al., 2017), sarcoplasmic reticulum-specific calcium cycling (Wang et al., 2014), metabolic status (Mercader et al., 2012; Wengrowski et al., 2014; Kuzmiak-Glancy et al., 2015; Jaimes et al., 2016a; Garrott et al., 2017), the efficacy of ablation therapies (Swift et al., 2014), stem-cell engraftment (Costa et al., 2012; Shiba et al., 2012; Filice et al., 2020), cardiotoxicity (Jaimes et al., 2019b), and the pathobiology of heart failure (Spragg and Kass, 2006; Ng et al., 2014; Zasadny et al., 2020). Due to differences in scientific inquiry, each optical mapping lab is unique in their focus and imaging approach. This manifests in the use of cameras with varying acquisition speeds (100–10,000 frames per second) and sensor size (100 × 100 to 2,000 × 2,000 pixels), simultaneous or interleaved light sources, and a variety of cardiac tissue preparations or distinct dye combinations with differing signal-to-noise profiles. Accordingly, individual laboratories often develop customized software tools to analyze the large volumes of spatial and temporal data produced by optical experiments (Laughner et al., 2012; Doshi et al., 2015; O’Shea et al., 2019). As a result, access to software analysis tools is limited and can impede the adoption of optical mapping techniques to the broader research community.

**FIGURE 1:**
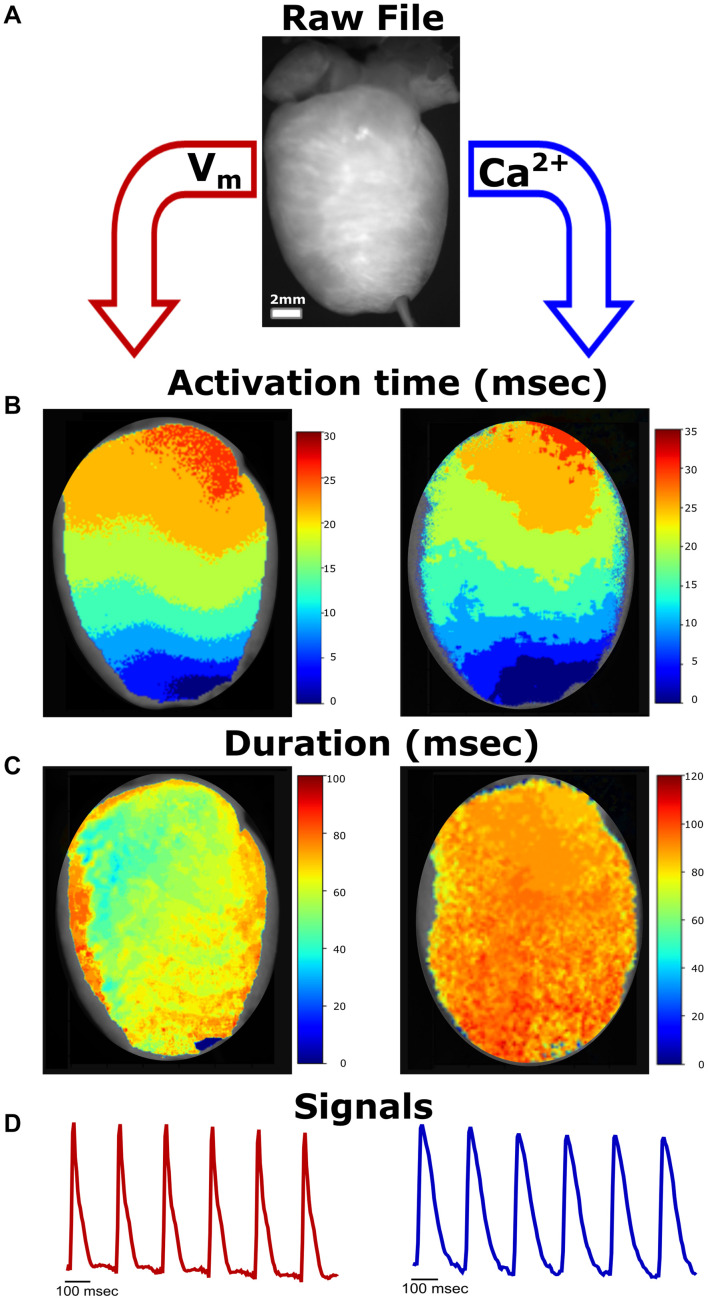
Simplified Workflow. **(A)** Representative raw image file from a Langendorff-perfused rat heart study (*.tif stack). Image properties (e.g., frame rate) are defined by the user. In our experimental setup, voltage and calcium signals can be analyzed from the same heart preparation. **(B)** Activation maps generated from raw image file demonstrate propagation away from the pacing electrode at the apex (left—voltage, right—calcium). **(C)** Generated action potential duration (left) and calcium transient duration (right) maps. **(D)** Representative traces from the raw image file.

To increase accessibility to optical mapping approaches, a few software applications have been released as open-source packages with freely available code, installation instructions, and user manuals [e.g., Rhythm (Laughner et al., 2012; Gloschat et al., 2018), Orca (Doshi et al., 2015), ElectroMap (O’Shea et al., 2019), and Cosmas (Tomek et al., 2021)]. However, utilization of these analysis tools can be hindered by necessary software expertise, dependence on a programming language that is cost prohibitive, or significant investment in commercial software (e.g., Optiq—Cairn Research). To address these hurdles, we developed a new software solution with a condensed workflow (Figure 1) to analyze cardiac optical mapping data quickly and accurately. Notably, the presented software package is controlled by a graphical user interface, which can facilitate its use by individuals with limited data analysis expertise. The described software is aptly named “*KairoSight*” in reference to the Greek word for “opportune time” (Kairos) and the ability to “see” voltage or calcium signals acquired from cardiac tissue. Since this software package uses an open-source platform (Python), it does not require commercial or licensed components. In the described studies, we demonstrate the utility of our software application using experimental datasets collected from mammalian hearts (rat, guinea pig, swine) stained with RH237 (transmembrane voltage) and Rhod-2, AM (intracellular calcium), as well as hiPSC-CM stained with FluoVolt (membrane potential) and Fluo-4, AM (calcium indicator). We also demonstrate cardiac responsiveness to ryanodine (ryanodine receptor modulator) and isoproterenol (beta-adrenergic agonist)—two agents commonly used to monitor perturbations in calcium cycling—and highlight regional differences after an ablation injury. By reducing complexities associated with data analysis, *KairoSight* equips investigators with the necessary tools to evaluate cardiac physiology and pathophysiology without requiring dedicated computer science expertise or proprietary software.

## Materials and Methods

### Acquisition and Generation of Optical Datasets

Animal procedures were approved by the Institutional Animal Care and Use Committee of the Children’s Research Institute and followed the National Institutes of Health’s Guide for the Care and Use of Laboratory Animals. All animals were euthanized by exsanguination under anesthesia following heart excision, as previously described in detail (Jaimes et al., 2019a; Swift et al., 2019). Experimental data were acquired from isolated, intact hearts that were maintained on a temperature-controlled (37°C), constant-pressure (70 mmHg) Langendorff-perfusion system. To reduce motion artifact, Krebs-Henseleit perfusion buffer was supplemented with 10-15 μM (−/−) blebbistatin (Cayman Chemical) (Fedorov et al., 2007; Swift et al., 2012), or 10 mM 2, 3-butanedione monoxime (BDM; Sigma-Aldrich). Intact heart preparations were loaded with a calcium indicator dye (Rhod-2, AM: 100 μg rat, 100 μg guinea pig, 400 μg swine), which was added and allowed to stabilize for 10 min, followed by a potentiometric dye (RH237: 62 μg rat, 62 μg guinea pig, 248 μg swine). The epicardial surface was illuminated with filtered LED light (535 ± 25 nm), and emitted fluorescent light was split through a dichroic mirror (660 + nm) and directed through high-transmission filters to separate the V_m_ (710 nm long pass) and Ca^2+^ (585 ± 40 nm) signals. These simultaneous recordings were projected onto a single sensor with 2,016 × 2,016 pixel resolution and a pixel size of 11 × 11 μm (pco.dimax cs4), using an optosplit (Cairn Research) (Jaimes et al., 2019a; Swift et al., 2019). Adequate dye loading and signal detection was verified during the experiment using Fiji, an open-source image analysis software tool (Schindelin et al., 2012). Image stacks acquired from experimental data were saved in TIFF format (^∗^.tif) for subsequent analysis.

Cryopreserved hiPSC-CMs (iCell cardiomyocytes; Cellular Dynamics International) were thawed and plated onto fibronectin-coated cover glass (∼60,000 cells/cm^2^), as previously described. Cells were maintained under standard cell culture conditions (37°C, 5% CO_2_) in iCell cardiomyocyte maintenance medium (Cellular Dynamics International). Three days after plating, hiPSC-CMs formed a confluent monolayer, and were stained with either a potentiometric dye (FluoVolt; 1:1,000 dilution) or a calcium indicator dye (Fluo-4, AM; 10 μM) (Thermo Fisher Scientific) for 45 min at room temperature. Cell monolayers were then washed in dye-free Tyrode salt solution. Pace-induced action potentials and calcium transients were acquired at room temperature using a Nikon TiE microscope system, equipped with 470 nm excitation LED (SpectraX, Lumencor), 505–530 nm emission filter, and Photometrix 95B sCMOS back-illuminated camera. Cardiac monolayers were paced using field stimulation (monophasic, 10 ms pulse width, 1.5x threshold voltage, 0.3–0.7 Hz frequency).

### Software Development, Installation, and Image Analysis

*KairoSight* source code was written in the Python programming language (Python 3.8). Files related to the user interface elements and layout were generated in Qt Designer and converted to ^∗^.py python script files, suitable for the PyQt library used to give interface elements their functionality. When available and appropriate, methods from the standard Python libraries were used for data handling and analysis. Steps were taken to minimize user input and error by taking advantage of existing optimized numerical libraries like NumPy, scikit-image, and SciPy, Python’s core scientific computing library. The software is freely available to download as source code from https://github.com/kairosight/kairosight-2.0, which can be run and edited in either the Spyder IDE or from the command prompt using the Anaconda distribution of Python. For convenience, an installation video, graphical user interface usage video, README file, and sample image stacks (spatially binned due to file size limitations) are provided on GitHub.

*Kairosight* processes optical data by first importing an image stack and then applying the following steps (Supplementary Figure 1): (1) Image Properties (e.g., acquisition speed, cropping), (2) Processing (e.g., masking, spatial and temporal filtering, detrending, and signal normalization), and (3) Analysis (e.g., isolate action potential or calcium transient signal, compute relevant timepoints and durations, export results as pixel-wise values or pseudo-color maps). Data can be exported after the Analysis step as either pixel signals (^∗^.csv), or optical maps (^∗^.png).

#### Step 1—Image Properties

In the user interface, an image stack was imported, and key properties of the dataset were provided by the user [frame rate (fps), image scale (px/cm), and image type (V_m_ or Ca^2+^)]. When necessary, signal intensities were inverted along a central value to assure the analysis of relatively positive deflections from baseline intensities, based on the dye and filter combination. For our experimental dataset, upright signals were analyzed for calcium, while inverted signals were analyzed for voltage. An area of interest was isolated by cropping and rotating—which reduced the analysis of irrelevant pixels.

#### Step 2—Processing

Background regions or non-fluorescent tissue areas were masked to isolate pixels of interest. The default algorithm, Otsu, generates a threshold value based on Otsu’s Method (Otsu, 1979; Lee et al., 2017) and classifies each pixel as “foreground” or “background.” Thresholding uses an operator defined percentage value (between 0.0 and 1.0) and includes that percentage of pixels as sorted by their intensity in the mask, and uses an operator defined kernel size to perform morphological closing, opening, and dilation to smooth the mask. Random Walk (Grady, 2006) uses an Otsu approach to label pixels according to user-defined values. An algorithm solves diffusion equations initiated at defined pixels to label pixels of similar value, wherein unknown pixels are assigned the label they have the highest probability to reach during diffusion. Mean calculates the mean pixel intensity and labels all pixels below that threshold as background, and all pixels above the threshold as foreground (Glasbey, 1993). Using the first frame, background pixels were masked and assigned an intensity value of zero for the entire stack, and the dimensions of the stack were preserved throughout. Whether automated or user-guided, masking effectively isolated the brightest continuous region of a cardiac preparation independent of dynamic changes in the fluorescent signal from action potentials or calcium transients. Image stacks can also be convolved with a kernel, to blur or smooth the image by placing greater weight on pixels closer to the center pixel as described by a Gaussian curve, while minimizing spatial broadening of the optical signal (Laughner et al., 2012).

Voltage and calcium dyes often exhibit a small fractional change in fluorescence, which can result in a modest signal-to-noise ratio that impedes signal analysis without additional processing steps. Fluorescence signals regularly contain spatial noise, temporal noise, and baseline drift due to photobleaching. High-frequency temporal noise can be minimized, when needed, using a bidirectionally applied Butterworth filter with a user defined cutoff (e.g., 100 Hz). Spatial “salt-and-pepper” noise can be reduced by convolution with an image kernel filter (e.g., built-in box blur with adjustable kernel size). Signal drift can be removed by calculating and subtracting a least square fit polynomial curve of user-specified order for each pixel. Pixel intensity can be converted from arbitrary units of fluorescence (AUF) to normalized intensity (0 for minimum to 1 for maximum). Normalization from 0 to 1 scales all pixels to the same dynamic range, which facilitates subsequent workflow steps.

#### Step 3—Analysis

Analysis steps were performed to produce imaging results and maps from either individual or ensembled signals from suitable pixel(s). Specific timepoints and durations within the action potential or calcium transient signals were detected and calculated. The “start time” marker was placed prior to the upstroke of the signal and the “end time” marker was placed following the peak of the transient (activation time measurement) or following the return of the transient to baseline (duration time measurement; see Supplementary Figure 1). To identify inflections of interest, 1st and 2nd derivatives of each fluorescent signal were generated and smoothed. Maxima and minima of those derivations were used to identify the inflection points (timepoints) of interest within each action potential or calcium transient. For duration maps, the maximum expected time interval and the desired duration percentage (e.g., APD_80_) was defined by the user.

Between the baseline period and the peak time, the activation time (dFmax) of the action potential or calcium transient were identified. Downstroke (dFmin) and end time (2nd dF2max) were identified after each peak time, but before the subsequent baseline period began (Laughner et al., 2012). Identification of fundamental time points enabled further computation of optical signal features (e.g., action potential or calcium transient durations (APD or CaD: time from activation to any% of Fmax), diastolic interval (DI: time from APD_80_ or CaD_80_ to the subsequent activation), the time for an action potential or calcium transient to reach 1-1/e amplitude (τ: mono-exponential decay time (Jaimes et al., 2016b)).

In addition to the temporal results derived from optical signals, spatial analysis was important for identifying tissue heterogeneities, voltage and calcium concordance, and the directionality of electrical and/or calcium wavefront propagation. Functional activation patterns were displayed using isochronal maps, in which an activation time was assigned to each pixel and presented simultaneously. Similarly, spatial distributions of durations (APD, CaD) were displayed in scaled maps.

### Exporting Data

Images and text were exported through the user interface. A single frame or entire image stack was exported as a ^∗^.tif and a single frame or any generated map was exported as a ^∗^.png. Further, generated maps or corresponding temporal, spatial, or statistical results were exported as ^∗^.csv. Signal data was exported to obtain an (x,y) plot of action potential or calcium transient values. An array of fluorescence data was exported for each ROI over time, including intensity signals, analysis results (all values or mean ± SD) or calculated/mapped values as ^∗^.csv.

### Statistical Analysis

**FIGURE 2:**
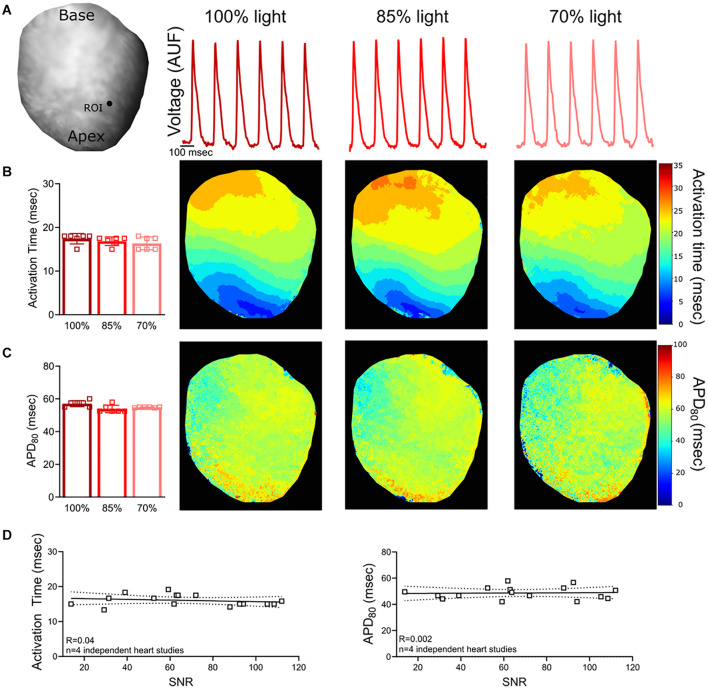
Voltage Signal-to-Noise Ratio (SNR). **(A)** Representative rat heart loaded with voltage dye, RH237. Action potentials were analyzed from the same region of interest (ROI) on the epicardial surface (*n* = 6 action potentials, same heart with diminished light illumination). **(B)** No measurable change in activation time was noted when the excitation light intensity and SNR was reduced (*n* = 6 action potentials, same heart). **(C)** Calculated APD_80_ values also remained consistent (*n* = 6 action potentials, same heart). **(D)** Relationship between SNR and action potential measurements (*n* = 4 optical signals collected from *n* = 4 independent heart studies). Epicardial dynamic pacing (160 ms PCL) from the apex of the left ventricle.

**FIGURE 3:**
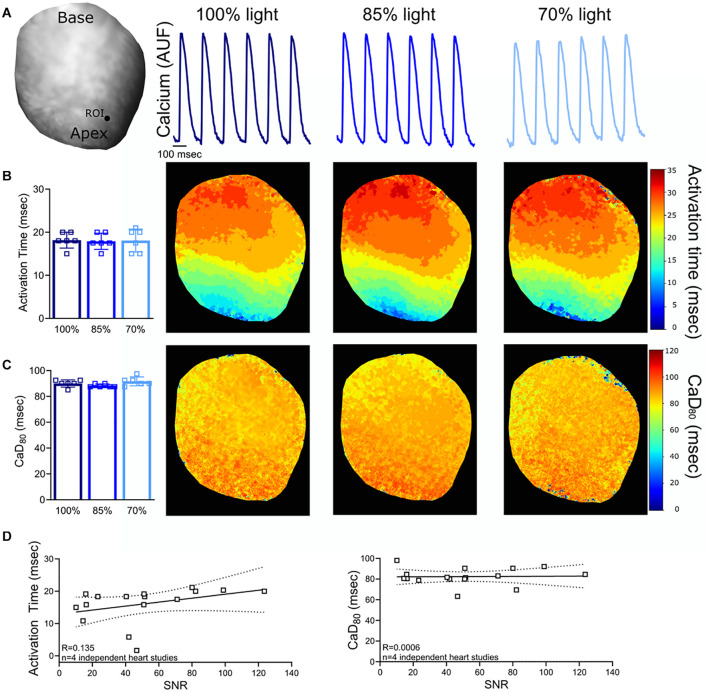
Calcium Signal-to-Noise Ratio (SNR). **(A)** Representative rat heart loaded with calcium dye, Rhod-2, AM. Calcium transients were analyzed from the same region of interest (ROI) on the epicardial surface (*n* = 6 calcium transients, same heart with diminished light intensity). **(B)** No measurable change in activation time was noted when the excitation light intensity and SNR was reduced (*n* = 6 calcium transients, same heart). **(C)** Calculated CaD_80_ values also remain unchanged (*n* = 6 calcium transients, same heart). **(D)** Relationship between SNR and calcium transient measurements (*n* = 4 optical signals collected from *n* = 4 independent heart studies). Epicardial dynamic pacing (160 ms PCL) from the apex of the left ventricle.

**FIGURE 4:**
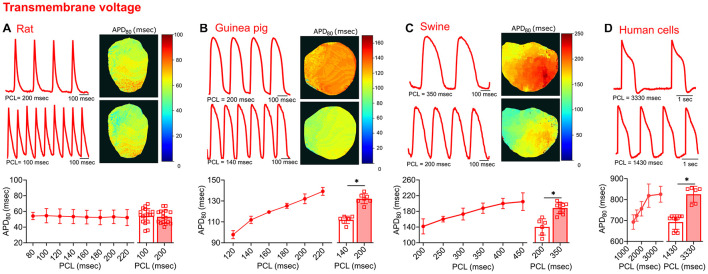
Transmembrane voltage measurements acquired from four different species. **(A)** Representative signals and APD_80_ maps from a rat heart paced at 200 and 100 ms PCL (top). Compiled restitution curve of APD_80_, in response to dynamic epicardial pacing (bottom left, *n* = 13–20 independent heart experiments). No change in APD_80_ was observed between 100 ms and 200 ms PCL (bottom right, *n* = 16–19 independent heart experiments). **(B)** Representative signals and APD_80_ maps from a guinea pig heart paced at 200 and 140 ms PCL (top). Compiled restitution curve of APD_80_, in response to dynamic epicardial pacing (bottom left, *n* = 5–7 independent heart experiments). Longer APD_80_ values observed at slower rates (200 vs. 140 ms PCL, *n* = 7 independent heart experiments). **(C)** Representative signals and APD_80_ maps from a swine heart paced at 350 and 200 ms PCL (top). Compiled restitution curve of APD_80_, in response to dynamic epicardial pacing (bottom left, *n* = 4–10 independent heart experiments). Longer APD_80_ values observed at slower rates (350 vs. 200 ms PCL, *n* = 6–10 independent heart experiments). **(D)** Representative signals from hiPSC-CM paced at 3330 and 1430 ms PCL (top). Compiled restitution curve of APD_80_, in response to field potential stimulation (bottom left, *n* = 6–15 independent cell preparations). Longer APD_80_ values observed at slower rates (3330 vs. 1430 ms PCL, *n* = 6–10 independent cell preparations). Values reported as mean ± SD. *p < 0.05, as determined by an unpaired Student’s *t*-test (two-tailed).

**FIGURE 5:**
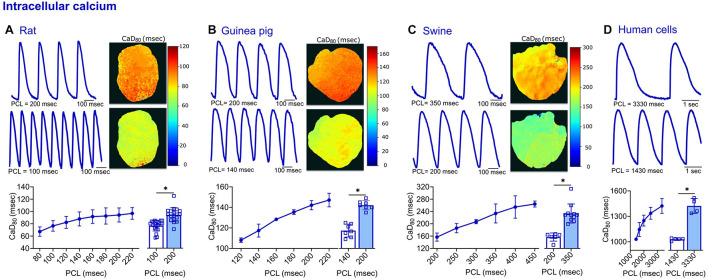
Calcium transient measurements acquired from four different species. **(A)** Representative signals and CaD_80_ maps from a rat heart paced at 200 and 100 ms PCL (top). Compiled restitution curve of CaD_80_, in response to dynamic epicardial pacing (bottom left, *n* = 11–19 independent heart preparations). Longer CaD_80_ values observed at slower rates (200 vs. 100 ms PCL, *n* = 18–21 independent heart preparations). **(B)** Representative signals and CaD_80_ maps from a guinea pig heart paced at 200 and 140 ms PCL (top). Compiled restitution curve of CaD_80_, in response to dynamic epicardial pacing (bottom left, *n* = 5–7 independent heart preparations). Longer CaD_80_ values observed at slower rates (200 vs. 140 ms PCL, *n* = 7 independent heart preparations). **(C)** Representative signals and CaD_80_ maps from a swine heart paced at 350 and 200 ms PCL (top). Compiled restitution curve of CaD_80_, in response to dynamic epicardial pacing (bottom left, *n* = 3–11 independent heart preparations). Longer CaD_80_ values observed at slower rates (350 vs. 200 ms PCL, *n* = 6–11 independent heart preparations). **(D)** Representative signals from hiPSC-CM paced at 3330 and 1430 ms PCL (top). Compiled restitution curve of CaD_80_, in response to field potential stimulation (bottom left, *n* = 4–5 independent cell preparations). Longer CaD_80_ values observed at slower rates (3330 vs. 1430 ms PCL, *n* = 4–5 independent cell preparations). Values reported as mean ± SD. *p < 0.05, as determined by an unpaired Student’s *t*-test (two-tailed).

**FIGURE 6:**
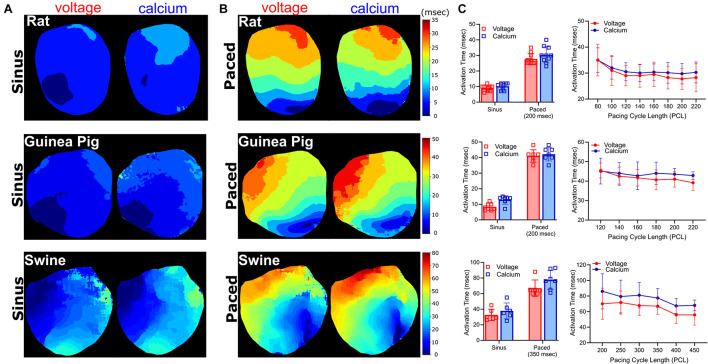
Activation time measurements. Voltage and calcium activation maps generated from excised Langendorff-perfused hearts, during **(A)** sinus and **(B)** epicardial dynamic pacing applied to the left ventricle (rat: 200 ms, guinea pig: 200 ms, swine: 350 ms PCL). **(C)** Left: Average activation time delay during sinus rhythm, compared with dynamic epicardial pacing (*n* = 10 rat, *n* = 7 guinea pig, *n* = 6–8 swine independent heart preparations). Right: Activation time restitution curves for rat (*n* = 4–8), guinea pig (*n* = 6–7), and swine (*n* = 3–6 independent heart preparations).

**FIGURE 7:**
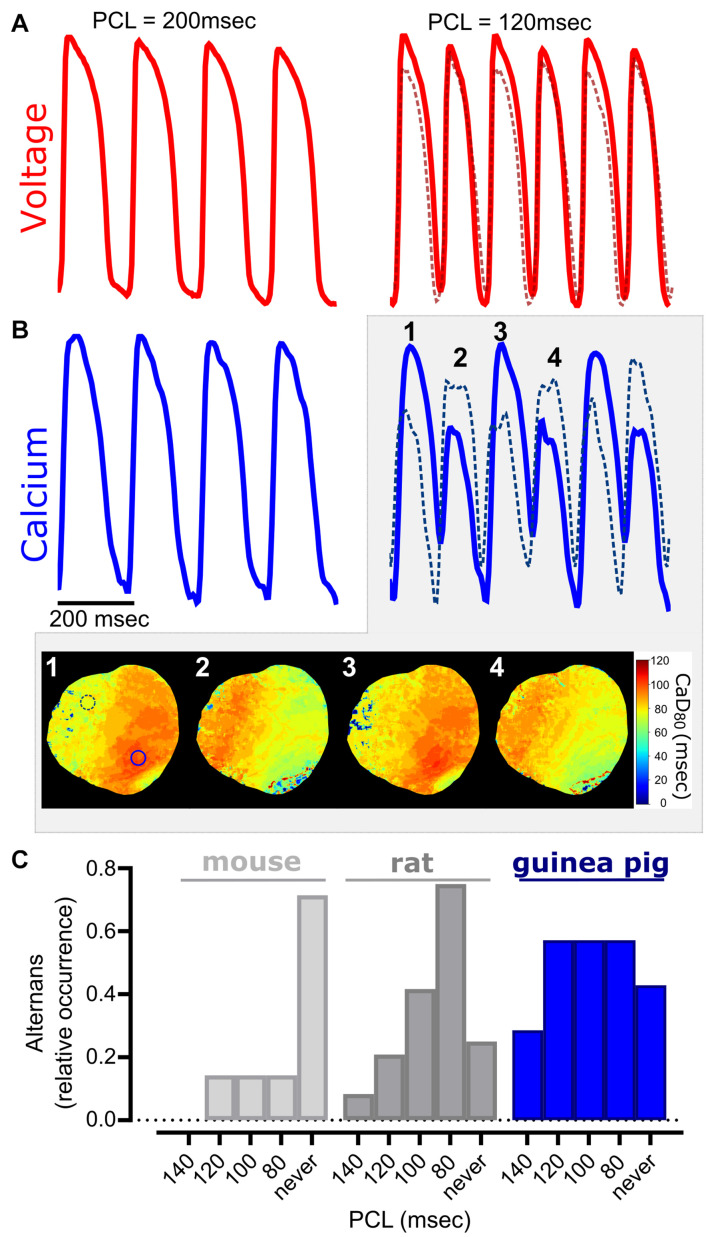
Cardiac alternans occur at faster pacing cycle lengths. **(A)** Representative optical action potentials, and **(B)** calcium transients from a Langendorff-perfused guinea pig heart. Slower PCL (200 ms) produces uniform action potentials and calcium transients (left), while a faster PCL (140 ms) results in cardiac alternans (right). Traces were acquired from two different regions of interest (solid line—left ventricle, dotted line—right ventricle) and discordant alternans were readily observable in the calcium signal. Four sequential beats show regional differences in alternans in calcium duration maps. **(C)** Relative occurrence of cardiac alternans in different species (mouse, rat, guinea pig). Epicardial dynamic pacing applied to the left ventricle for all studies (*n* = 5–18 independent heart studies per species).

**FIGURE 8:**
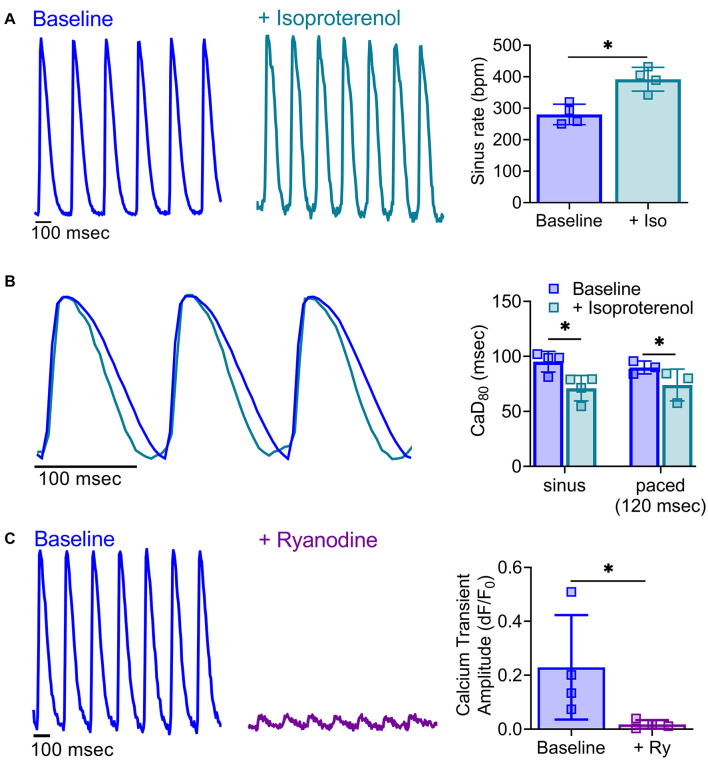
Impact of pharmacological agents on calcium handling. **(A)** Representative calcium transients acquired from a Langendorff-perfused rat heart at baseline and after acute treatment with isoproterenol (30 nM). Isoproterenol increased sinus rate (right, *n* = 4 independent heart studies, *p < 0.05 as determined by two-tailed Student’s *t*-test). **(B)** Shorter calcium transient duration time in response to isoproterenol application was readily observable in calcium signals from a Langendorff-perfused rat heart during sinus rhythm and with external pacing for rate correction (120 ms PCL, *n* = 3–4 independent heart studies, *p < 0.05 as determined by two-way ANOVA with multiple comparisons testing). **(C)** Representative calcium transients from a Langendorff-perfused rat heart at baseline and after acute treatment with ryanodine (5 μM), during external pacing (160 ms PCL). Ryanodine diminished calcium transient amplitude (right; *n* = 4 independent heart studies, *p < 0.05 as determined by two-tailed Student’s *t*-test). Values reported as mean +SD. BPM = beats per minute, dF/F0 = change in fluorescence over baseline.

Results were reported as mean ± standard deviation. Differences between group means were determined using two-tailed Student’s *t*-test or analysis of variance (GraphPad Prism). Significance was defined as a *p*-value < 0.05, or an adjusted *p*-value (*q* < 0.1) after correction for multiple comparisons using two stage linear step-up procedure to control the false discovery rate (0.1), as described by Benjamini et al. (2006). Significance was denoted in the figures with an asterisk (^∗^). Replicates were defined in each figure legend; independent optical signals were used from the same heart preparation (Figures 2A–C, 3A–C signal-to-noise data), and independent heart preparations were used for other measurements (Figures 4–8).

## Results

### *KairoSight* Workflow and User Interface

To provide a more accessible tool for optical mapping data analysis, we developed a novel, open-source software package with an intuitive user interface (Supplementary Figure 1) that directs users through a linear workflow (example shown in Figure 1). Image processing was accomplished by importing an image stack and applying the following steps: (1) Image Properties, (2) Processing, and (3) Analysis. The *KairoSight* interface used a workflow with very few input options, which minimized user error, maximized computational resources, and enabled new users to easily understand the methods to implement data analysis steps with accuracy. To illustrate the Image Properties step, cropping and masking were applied to an experimental dataset in Figure 1.

### Analysis of Transmembrane Voltage and Intracellular Calcium

**FIGURE 9:**
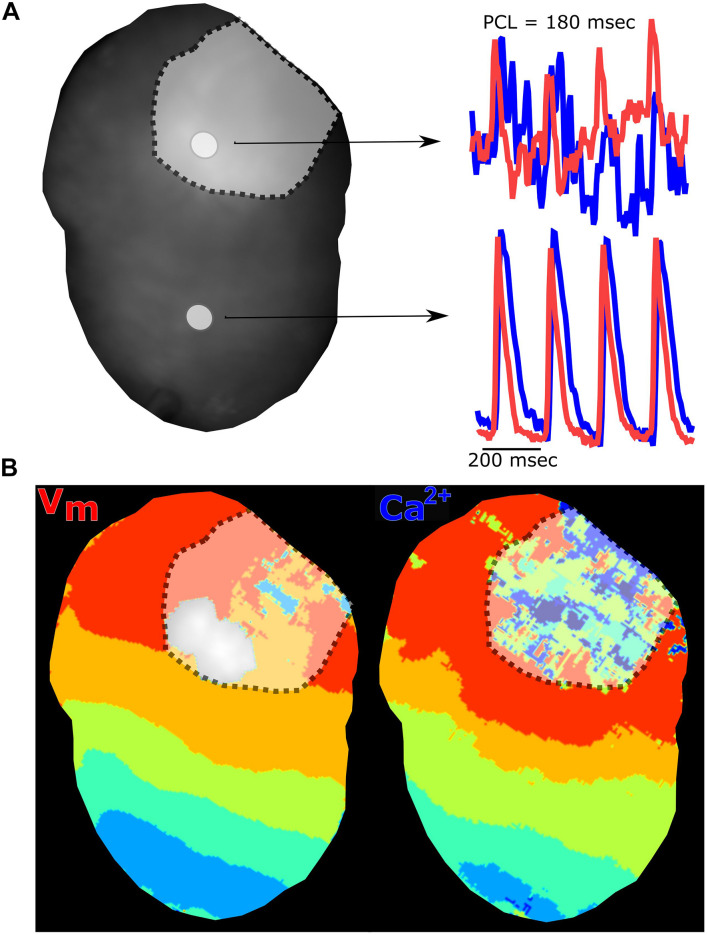
Myocardial injury via radiofrequency ablation diminishes electrical and calcium signals. **(A)** Langendorff-perfused rat heart after radiofrequency ablation showing two regions of interest and corresponding traces. The voltage signal (red) and calcium signal (blue) are collected from the ablation site (denoted with dotted line) and non-ablated tissue. **(B)** Voltage and calcium isochronal maps display activation time across the heart, with disruption at the site of ablation injury. Epicardial dynamic pacing from the apex of the heart.

Multiparametric optical signals were acquired from Langendorff-perfused rat, guinea pig, and swine hearts, and hiPSC-CM, which were stained with fluorescent dyes. Using the cropping feature, the user isolated areas of interest and specified the type of signal (voltage—inverted signal, or calcium—upright signal) from the images loaded onto *KairoSight*. Activation maps, action potential, or calcium transient duration maps (Figures 2–9), as well as statistical results (e.g., action potential and calcium transient duration, amplitude, activation time, transient decay tau) were generated. The software identified and analyzed optical voltage and calcium signals from multiple species (Figures 4, 5, 7), with variations in signal shape and pacing frequency.

### Suboptimal Signal-to-Noise Ratio

To confirm the consistency of the signal analysis software in the event of imperfect signals, experimental data was analyzed with varying degrees of noise. Optical signals were collected from hearts loaded with a voltage sensitive dye (RH237) and a calcium dye (Rhod-2, AM). The signal-to-noise ratio (SNR; calculated as the signal amplitude divided by the standard deviation of the baseline during the diastolic interval) was decreased by reducing LED illumination on the surface of the heart. Reducing LED illumination decreased voltage signal SNR (*100% light*: SNR 118.1 ± 19.1, *85% light*: 104.9 ± 10.6, *70% light*: 85.0 ± 4.5) and calcium signal SNR (*100% light*: SNR 118.9 ± 19.2, *85% light*: 104.0 ± 9.8, *70% light*: 82.3 ± 6.2). *Kairosight* was used to analyze corresponding voltage (Figures 2A–C) and calcium signals (Figures 3A–C) within the same heart, wherein only the degree of LED illumination was altered. Activation times for voltage (*100% light*: 17.4 ± 1.2, *85% light*: 16.8 ± 1, *70% light*: 16.3 ± 1.5 ms) and calcium (*100% light*: 18.2 ± 1.8, *85% light*: 17.9 ± 1.9, *70% light*: 18.1 ± 1.1 ms) were calculated from the middle of the epicardial surface, along with action potential duration time at 80% repolarization (APD_80_ *100% light*: 57.1 ± 1.9, *85% light*: 53.8 ± 2.3, *70% light*: 54.8 ± 0.3 ms) and calcium transient duration at 80% reuptake (CaD_80_: *100% light*: 90 ± 2.7, *85% light*: 88.3 ± 1.3, *70% light*: 91.7 ± 3.4 ms); values were computed from *n* = 6 optical signals within the same heart. Experiments were then repeated (*n* = 4 independent heart preparations) under varying degrees of illumination to alter the SNR (Figures 2D, 3D). We noted deviations in the activation time for the calcium signal (“calcium release”) when SNR < 50, but calcium transient duration measurements remained consistent.

### Multi-Species Analysis

To test the flexibility of the software in analyzing various signal morphologies, tissues from four source species were analyzed. Transmembrane voltage signals were collected from the hearts of rats, guinea pigs, and swine, as well as cultured hiPSC-CM layers. In comparing the morphology of the electrical and calcium traces between species, varying differences in the optical signals were detected (Figures 4, 5). Rat action potential and calcium transient optical signals aligned with examples in the literature (Walton et al., 2013; Jaimes et al., 2016b; Zasadny et al., 2020). The corresponding electrical restitution curve showed little change when the dynamic pacing cycle length (PCL) was decremented from 200 to 100 ms (Figure 4A), however, the CaD_80_ shortened by 18.8% at the faster pacing rate (Figure 5A). Guinea pig signals and restitution values mirrored those reported in the literature (Guo et al., 2008; Lee et al., 2012), as well as those modeled by Edwards and Louch (2017). As expected, duration times were longer in the guinea pig heart, compared to the rat. Specifically, the APD_80_ and CaD_80_ shortened by approximately 15.2 and 17.4%, respectively, as the PCL was decremented from 200 to 140 ms (Figures 4B, 5B). Likewise, swine APD_80_ and CaD_80_ exhibited even slower dynamics, in agreement with previous studies (Bourgeois et al., 2011; Walton et al., 2013; Lee et al., 2017). Decrementing the PCL from 350 to 200 ms shortened the APD_80_ by 25.5% (Figure 4C) and the CaD_80_ by 30.3% (Figure 5C). Although not directly comparable to an intact heart preparation, hiPSC-CM were also imaged and analyzed as proof of concept. Decrementing the PCL from 3,330 to 1,430 ms shortened the APD_80_ by 16.1% (Figure 4D) and CaD_80_ by 27.6% (Figure 5D).

### Activation Time Measurements

Action potential and calcium transient activation maps were generated for rat, guinea pig, and swine hearts during both sinus rhythm and in response to dynamic epicardial pacing (Figure 6). As expected, activation time measurements were faster during sinus rhythm (often occurring in the right ventricle before the left ventricle) and slower in response to external pacing. In the rat heart, activation time was 8.9 ± 1.9 ms (V_m_) and 10.2 ± 2.1 ms (Ca^2+^) during sinus rhythm (Figure 6A), which increased to 27.7 ± 3.5 ms (V_m_) and 30.4 ± 5.2 ms (Ca^2+^) with pacing (200 ms PCL; Figures 6B,C). Similar observations were noted in larger species; in the guinea pig heart activation time was 18.8 ± 5 ms (V_m_) and 19.9 ± 6.3 ms (Ca^2+^) during sinus rhythm (Figure 6A), which increased to 29.4 ± 3.4 ms (V_m_) and 31.9 ± 4.8 ms (Ca^2+^) with pacing (200 ms PCL, Figures 6B,C). Piglet heart activation time was 32.7 ± 7.3 ms (V_m_) and 37.8 ± 10.2 ms (Ca^2+^) during sinus rhythm (Figure 6A), which increased to 67.0 ± 10.5 (V_m_) and 77.4 ± 12.2 ms (Ca^2+^) with pacing (350 ms PCL, Figures 6B,C).

### Cardiac Alternans and Demonstration of Regional Heterogeneity

Calcium flux plays a central role in excitation-contraction coupling, wherein perturbations in calcium handling (e.g., alternans, calcium leak from the sarcoplasmic reticulum) are associated with an array of cardiac dysfunctions, both electrical and mechanical (Weiss et al., 2011; Wang et al., 2014; Kanaporis and Blatter, 2015; Johnson et al., 2019; Sutanto et al., 2020). Calcium alternans are a potentially deleterious physiological oscillation, which are observable as alternating amplitude fluctuations in optical signals. The latter has been mechanistically linked to T-wave alternans that can segue into lethal ventricular arrhythmias (Weiss et al., 2011; Ng, 2017). Experimentally, cardiac alternans have been more commonly observed at faster heart rates and/or cooler temperatures (Egorov et al., 2012; Millet et al., 2021); accordingly, we utilized burst pacing to induce alternans in isolated, intact hearts (Figures 7A–C). Although not observed at slower pacing rates (200 ms PCL), intact guinea pig hearts displayed both APD alternans and calcium transient amplitude alternans at faster rates (120 ms PCL, Figures 7A,B). Further, calcium transient duration maps revealed spatially discordant alternans at faster rates, in which two regions of the tissue (right vs. left ventricle) were out of phase. When considering the occurrence of alternans between species, we observed variable susceptibility with increasing pacing frequency (relative occurrence at 100 ms PCL: 14% in mice, 42% in rats, and 57% in guinea pigs; Figure 7C).

### Experimental Techniques to Demonstrate Responses to Pharmacological Agents and Myocardial Injury

Pharmacological agents are frequently employed to identify the mechanisms responsible for cardiac responses. As one example, isoproterenol is commonly used to induce heart failure through its positive chronotropic effects (Balakumar et al., 2007; Greer-Short and Poelzing, 2015; Chang et al., 2018). Using Langendorff-perfused rat hearts, we demonstrated that acute isoproterenol exposure (30 nM) results in a 40% increase in sinus rate (280.3 ± 32.4 baseline vs. 392.0 ± 37.7 BPM with isoproterenol), and 17.7% shortening of CaD_80_ with dynamic pacing at 120 ms PCL (89.9 ± 5.9 baseline vs. 74 ± 14.4 ms with isoproterenol; Figures 8A,B). Further, ryanodine (5 μM) modulates calcium release from the sarcoplasmic reticulum, and is often used to demonstrate signal specificity in dual optical mapping studies (Choi and Salama, 2000; Jaimes et al., 2019a). Using *KairoSight*, we demonstrated a marked 92% reduction in the calcium transient amplitude with minimal effects on optical action potentials collected from intact rat hearts (Figure 8C). Optical mapping studies are often used to monitor spiral wave formation and re-entry arrhythmias (Moe and Jalife, 1977; Pumir et al., 2005), which may occur due to tissue heterogeneities from a myocardial infarction or incomplete cardiac ablation. To demonstrate the utility of our optical mapping approach and data analysis, an epicardial radiofrequency ablation was applied to mimic the physiological environment that can promote such arrhythmias (Mercader et al., 2012). Optical signals were collected before and after radiofrequency ablation, and the analyzed signals show a typical pattern of electrical activity and calcium fluctuations outside the ablated area (Figure 9). Within the ablated area, we observed irregular and diminished signals from both the voltage and calcium signals. Accordingly, optical signal analysis via *KairoSight* can be applied to other applications focused on pharmacological treatment, tissue injury, or pathophysiology.

## Discussion

Optical mapping is commonly used in cardiac research applications, as it allows one to resolve the electrical and calcium oscillations that underly cardiac function and dysfunction (Jaimes et al., 2016b; George and Efimov, 2019). Nevertheless, there are important technical limitations and constraints that should be considered. Although optical mapping can be employed *in situ* (Lee et al., 2019; Martišienė et al., 2020)—this technique is more commonly applied *ex vivo*, using Langendorff-perfused heart preparations that are maintained with a crystalloid buffer. To avoid signal distortion, the motion of the heart is often suppressed using mechanical constraint or excitation-contraction uncoupling agents (Fedorov et al., 2007; Swift et al., 2012). In the latter, the secondary effects of uncoupling agents should be considered (e.g., reduced metabolic demand, altered electrical activity). Moreover, in Langendorff-perfused heart preparations, optical mapping is limited to the surface of the tissue and endocardial activity can only be surmised by what is “seen” on the epicardium. Similarly, measurements of activation time and conduction velocity are monitored in response to external pacing—rather than under sinus rhythm. Despite these limitations, optical mapping remains a powerful tool for studying the spatiotemporal dynamics of cardiac electrophysiology and excitation-contraction coupling.

Optical mapping techniques have evolved over the last few decades, with continuous advancements in voltage and calcium-sensitive dyes, light source and photodetector technology. As just one example, in 1975 the first fully digital camera took 23 s to record a 100 × 100 pixel image, while newly developed CMOS sensors have offered improved spatiotemporal resolution with acquisition speeds that can exceed 2,000 frames per second. Despite these immense technical improvements, data analysis remains an obstacle for new investigators entering the field of optical mapping, as many groups still develop their own custom software. To help address this barrier to entry, we have developed an accessible and functional software platform to prepare, process, and analyze optical action potentials and calcium signals collected from cardiac preparations. In the presented article, we document the utility of *KairoSight* in analyzing optical data sets collected under conditions of variable SNR, demonstrate species-specific differences in action potentials and calcium transients, showcase cardiac responses to varying pacing frequencies and/or pharmacological agents, and highlight tissue heterogeneities in the context of discordant alternans and cardiac injury. Our open-source software was developed using Python, as it has consistently been ranked as a “top 10” programming language by industry analysts (Stephens, 2020). Due to its flexibility and widespread adoption, we fully anticipate the research community to utilize *KairoSight* for image analysis applications—and also modify and tailor this tool for their own research aims. For example, future updates to the software could include measurements of conduction velocity, automated detection of electrical or calcium alternans, and/or the generation of signal:noise ratio maps.

## Data Availability Statement

Datasets are available from the corresponding author on reasonable request.

## Ethics Statement

The animal study was reviewed and approved by Institutional Animal Care and Use Committee of the Children’s Research Institute.

## Author Contributions

BC, CG, LS, TP, DM, RJ, and NP performed the experiments and approved the manuscript. BC, CG, LS, and NP analyzed the data. BC, LS, and NP prepared the figures. BC, LS, DM, and NP drafted the manuscript. BC, LS, CG, RJ, DM, and NP conceived and designed the experiments. All authors contributed to the article and approved the submitted version.

## Conflict of Interest

The authors declare that the research was conducted in the absence of any commercial or financial relationships that could be construed as a potential conflict of interest.

## Publisher’s Note

All claims expressed in this article are solely those of the authors and do not necessarily represent those of their affiliated organizations, or those of the publisher, the editors and the reviewers. Any product that may be evaluated in this article, or claim that may be made by its manufacturer, is not guaranteed or endorsed by the publisher.

## Supplementary Material

Supplementary Figure 1 Example User Interface. Representative TIFF stack (^∗^.tif) loaded into the *KairoSight* interface, with “Image Properties” including frame rate defined by the user. During “Processing,” background was removed, pixels were box blurred 3 × 3, 100 Hz filter was applied, signal drift was removed, and traces were normalized (0–1). During “Analysis,” the start and end of a signal upstroke was defined by the user to measure activation time.

## Abbreviations

APD_80_: action potential duration at 80% repolarization
AUF: arbitrary units of fluorescence
Ca^2+^: intracellular calcium
CaD_80_: calcium transient duration at 80% reuptake
dF/F0: change in fluorescence over baseline
hiPSC-CM: human induced pluripotent stem cell-derived cardiomyocytes
PCL: pacing cycle length
SNR: signal-to-noise ratio
V_m_: transmembrane voltage.
