# Supplementary figures and images for: KairoSight: Open-Source Software for the Analysis of Cardiac Optical Data Collected From Multiple Species
Source: Front Physiol. 2021 Oct 29;12:752940. doi: 10.3389/fphys.2021.752940 (PMC8586513; doi:10.3389/fphys.2021.752940)

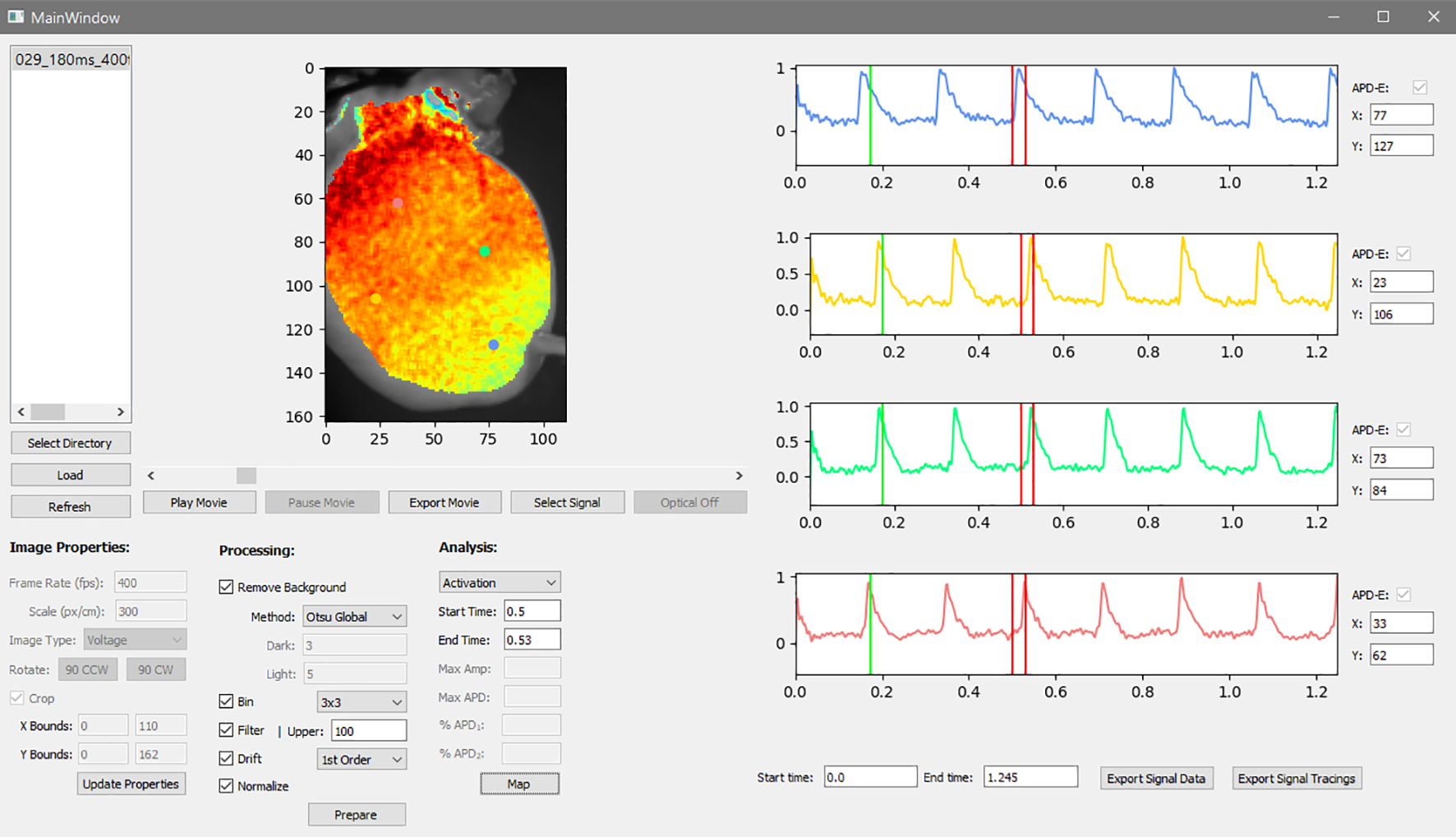

Supplement: Supplementary Figure 1 — Example User Interface. Representative TIFF stack (∗.tif) loaded into the KairoSight interface, with “Image Properties” including frame rate defined by the user. During “Processing,” background was removed, pixels were box blurred 3 × 3, 100 Hz filter was applied, signal drift was removed, and traces were normalized (0–1). During “Analysis,” the start and end of a signal upstroke was defined by the user to measure activation time. [file Image_1.TIF]
